# Essay on Cleft Palates

**Published:** 1878-01

**Authors:** 

**Affiliations:** North Adams.


### ARTICLE V.

### *Essay on Cleft Palates.*

Read before the Connecticut Valley Dental Society, at Greenfield, Mass., June 13th, 1877.

BY DR. DAVENPORT, OF NORTH ADAMS.

Mr. President and Gentlemen: Medicine, surgery and dentistry are all based upon anatomy, physiology, pathology and materia medica. Without anatomy, we should not know either the structure of a single tooth, or its connection with the jaw, gums, blood vessels, nerves, etc. Without physiology, we should know neither the natural uses and influences of the several parts just named, nor the relation of the teeth to the whole process of digestion, assimilation and nutrition.

As pathology bears the same relation to organized structures in an imperfect or diseased condition as physiology does to them in the natural condition, so, without a knowledge of it, neither the physician, surgeon or dentist could know anything of the origin, nature and tendencies of the diseases and defects he professes to treat. The materia medica, in its full scope, includes everything that can be made useful in the mitigation or removal of any of the ills to which man is liable.

These four branches of medical study (if no more) are fundamental, and no man can do full justice, practically, to the most limited specialty, without a thorough knowledge of them all.

Every member of this Association will acknowledge that a dentist should certainly understand the composition, structure and mode of development of the teeth, together with the causes that render their development defective, or induce in them disease and decay. A knowledge of these structures, whether chemically, anatomically or microscopically considered, involves a knowledge of the same structures in all other parts of the body. To understand the development of a tooth and its appendages from materials selected from the blood, involves a knowledge of the blood itself, and all the laws that govern the intricate processes of assimilation, nutrition and disintegration in living structures generally. Indeed there is not a living atom of our physical organization so isolated, that a knowledge of its structure, nutrition, disintegration and various morbid conditions can be obtained without developing all the essential facts and principles of anatomy, physiology and pathology.

It is an interesting field for physiological research, to understand every slight physiological act and observe every consequent histological condition, from the first appearance of the tooth gum, to its full and complete development. But this has not been the privilege of any one individual. Sufficient observation, however, has been made to determine the general conditions and the changes which occur duing the germination and growth of these organs.

The questions, then, which are of the most importance for our consideration in this connection, are: What are the causes which produce the abnormal conditions of these organs? Where does all this defective dental tissue which we see come from? And what is *the* remedy?

A very important inquiry connected with this subject is the question, In what sense, and how far, does the mother contribute during gestation to normal or abnormal dental structures; and, in this respect how far may abnormal results be prevented or removed? As maternity is the source and continuance of the nutritive pabulum during uterine gestation, and usually is (or, at least, should be) during the first few months of infantile life, the question arises, May and does not the mental and physical condition of the mother affect detrimentally the fœtal and in fantile aliment, both in regard to its quantity and quality?

If this question has its foundation in physiological truth, do not such indications call most imperatively upon the medical as well as the dental practitioner to furnish to mothers the instruction requisite to the full comprehension and imperative necessity of supplying themselves during this period with sufficient and appropriate nourishment to insure healthy fœtal nutrition? As a defective organization will be produced just in the ratio of a deficiency of those appropriate vegetable and mineral elements which enter into and contribute directly to the formation of fœtal structure, I have long thought that we are too much in the habit of looking on the surface of this subject, and standing with folded arms, and often with closed eyes, while millions upon millions of dental structures have gone down to premature graves.

"An ounce of prevention is worth more than a pound of cure." It was formerly supposed that the dental practitioner must take the dental organs as he finds them, and perform such surgical operations as he may consider best adapted to their preservation and health, entirely ignoring the great truth that it is the paramount of the physician and dentist to prevent disease. My impressions are, that our efforts have been directed more to remedying the effect than to removing the cause of this, I might say, national calamity —to cure rather than the prevention of disease. To find out the real cause of defective dentition and decay, we must consider the fundamental principles and laws of our existence. Physiology teaches us that all vegetable and animal bodies are composed of gases, liquids and solids of various kinds and properties. The body of a man is composed of some sixteen of these different substances, and they are governed by the same natural laws that control them in other forms or bodies. These substances are derived from the air, the earth and the water. Our common articles of food are known as simple and compound alimentary substances, and when taken into the system are converted into blood. The blood *should* contain all the requisite constituents for muscles, fat, bones, etc. But if any of the constituents of which these are formed are wanting in the food, there will be corresponding deterioration in those parts of organs which require them; for the body cannot originate a simple or a compound substance, nor can it appropriate any more of a constituent to an organ than it has received into the system for that purpose; hence the food which is designed to nourish all parts of the body should contain all the constituents of which the body is composed.

The body does not possess the power of converting one elementary substance into another; it can compound and appropriate substances to the various parts of the system, but it cannot originate them. If the mineral element which forms and sustains the hard portion is wanting, those organs will suffer just in proportion to that deficiency. The potter cannot fashion the bowl without the clay, neither can bone be formed without earthy matter. If there be an abundant supply of earthy matter to full meet the demands, then, instead of soft, chalky teeth, as we now have, they will be dense and firm in their teqture, and capable of re resisting decay, so long as a due proportion of mineral supplies are kept up. And under ordinary circumstances they will retain their integrity until their supplies are deminished or suspended. Some physiologists claim that the above is incorrect, and think that with a free use of a vegetable diet and animal food we at all times obtain sufficient nourishment for the dental organs, as well as for the whole bony structure of the body. Though we use white bread, and entirely discard the Graham bread and Graham mush of which there is so much said by a class of modern physiologists, claiming that the amazing amount of physical distress and dental disintegration which is met with on every hand does not occur so much from an insufficiency of bone element in our food, as the inability of our physical powers to make use of what we get. But whether your profession hold to the latter theory or to the former, I am not now so fully prepared to say as I shall be when I hear you comments on this paper. Personally I am inclined to the former, believing that when the food does not contain sufficient mineral matter, that the dental tissues may not only be defective, but we may also have congenital defects of the palate. In support of this it is said that ninety-nine per cent, of the lion cubs born in the Garden of the Zoological Society in Regent's Park, London, have cleft palates, so that they are unable to take nourishment, and consequently soon die. At the Dublin Zoological Gardens, however, there are very few such cases. This difference has been attributed to the difference in the food of the parent lions those in London being fed only on the flesh of large animals, so that they are unable to eat any of the bones; whereas in Dublin a goat twice a week is given them, which they eat bones and all. This provides the bone phosphates needed for the organization of the young animals. Prior to the adoption of this method of feeding at Dublin, malformation of the palate was as common there as it now is at London. This furnishes a fruitful field for thought, and is very suggestive on the subject in hand.

Having spoken of some of the causes which, to my mind, contribute largely to produce defective dentition and cleft palate, and having hinted at the remedy which must be adopted if we would avoid the causes upon hygienic principles which produce these abnormal development, I now propose briefly to speak of the remedy for congenital defects of the palate.

My experience in staphyloraphy has been too limited to enable me to engage your attention for any length of time, The object to be obtained by staphloraphy is to improve the articulation or speech, which is more or less defective in every case.

" Dr. Kingsley says that the closing of the fissure by surgical operation, no matter how skillfully performed, and no matter how perfectly the union is made, he believes to be in almost all cases a failure. The objects for which it is attempted are not abtained. I believe now almost every surgeon of eminence concedes the point, and also gives to the dental practitioner the credit of having proved that this operation is no longer necessary."

When we come to look into the mechanical process of articulation we find it produced by the voice or sound issuing from the glottis, and manipulated by the organs between that point and the lips inclusive, and, to a certain but very limited extent, the nostrils.

The palate, I believe, possesses a more powerful influence over articulation than any other organ, unless it be the tongue, and perhaps nearly or quite as much as that organ; for it is the tongue coming in contact with the palate, working against and articulation with it, thus breaking up the voice, that gives variety. And these sounds come to be understood as language. Now what we are called upon to do, is to supply the loss. I need not refer you to the attempt made in the olden times to fill this fissure by the use of unyielding substances. That subject is more or less familiar to you. The only substance that can be successfully used is elastic rubber. The palate, in order to articulate, must have the power to rise up until the passage to the nares is closed. I am not certion whether that closure is entirely due to the elevation of the palate, or whether, simultaneously, there is a contraction of the pharynx. I think, however, the pharynx meets the palate half way.

Perhaps it might be well for me to state here the reason why I have taken sufficient interest in this subject to bring it before you at this time. It is from the fact that I have for some time past had a patient under my care who has one of the largest fissures of the palate I have ever seen, it being complicated formerly with double hair-lip Consequently the ideas, suggestions and conclusions are all fresh from personal experience and treats made upon the patient, who is now wearing an appliance I made for her during the past winter.

I have found that the natural vela or palate is continually moving up and down, now closing the passage to the mouth, allowing the sound to pass through the nose, again opening and allowing the sound to pass both ways; then closing the passage to the nares, and allowing only a passage through the mouth; again closing the passage to the nares, and, in conjunction with the lips, allowing the sound to accumulate in the mouth, ready for an explosive sound, as in "B," the air accumulating in the mouth until the cavity is full, when, opening the lips, it makes "B" With "P" there is precisely the same action of every other organ, except there is not the accumulation of sound that "B" exhibits. "M" passes entirely through the nose; instead of the air accumulating, it continues to issue from the nose. In this operation the tongue remains quiet; the lips and the palate do the whole. I find by experiment that if the palate is defective so it cannot close the passage to the nares, and the sound issues there instead of accumulating in the mouth to form "B," it must form "M." We must, therefore, in making a substitute for the lost organ, make something that shall close the passage. This is the reason why I have found the operation a difficult one. The artificial palate must be made to accomplish the purpose, and be under the control of the muscles in such a manner that it may be elevated and dispressed by the remnants of the lost velum, and at the same time admit of the contraction of the sides of the fissure, or deglutition will be interfered with.

I found it greatly to my advantage, for the sake of experiment, as well as for convenience, to make my obturator and velum in separate pieces. I also have made several vela for this patient, varying each in some particular, and at the same time testing each, until I find that I am not able to go below the base of the uvula, and, owing to the lack of muscular power in the remnants of the natural velum, and also a weakness in the superior constrictor of the pharynx, I found it necessary, after coming to the base of the uvula, to reflect it back horizontally, so as to be out of the way of the food, and let it terminate in a thin and delicate edge—otherwise it would not close the nares. The posterior border is dropped a little, so that when the pharynx contracts it shall come against a curved edge. This I found necessary in order to produce perfect articulation, and also that it may not irritate the walls of the pharynx.

To make this instrument embrace the sides of the fissure, and at the same time provide for the contraction of the sides of the fissure in deglutition, has caused me great solicitude. I find in actual practice, that while all the edges must be delicate to prevent irritation, all the other parts must be just as firm as they can be made, and at the same time allow of an easy movement of the muscles. Firmness seems necessary, that the tongue, coming against it, may not drive it out of the way, but hold on long enough to get ready for the sound. In the letter "K" the sound is made by the hard, firm pressure of the palate and the tongue in conjunction with each other, the pharynx also acting with them. From all the information I can gather on this subject, I find we cannot look for immediate results in articulation, as all the muscles are trained to speak without the aid of the lost organs. Perfect articulation, then, can only be obtained by long persevering and continued practice. Since this patient has been wearing the appliance, I find a marked improvement in the articulation, and almost an entire absence of that nasal tone which always characterizes cases of this kind. I have found a difficulty with this patient which I do not understand. She does not seem to be able to distinguish the difference in the articulation of her own voice. This seems to be a serious barrier in regard to correcting the speech. Whether this is due to an absence of the Eustachian tubes or from any other cause I am not able to say. I have found, upon examination, that the Eustachian tubes (so far as I can judge) are present. Therefore my conclusion is, that the difficulty alluded to is due to some other cause. All the models of the lost organs I have duplicated in type metal, which I have brought for your examination. This is necessary, in order that the instrument may be duplicated at any time as soon as deterioration commences—this soft rubber being a perishable substance. And, from the experience I have had with soft rubber, we cannot expect the instrument to last more than from three months to a year. Hence the necessity of metal models, that duplicates can be furnished the patient at any time.—*Dental Miscellany.*

---

